# Gliosarcoma in patients under 20 years of age. A clinicopathologic study of 11 cases and detailed review of the literature

**DOI:** 10.1186/s12887-021-02556-9

**Published:** 2021-02-26

**Authors:** Nasir Ud Din, Hira Ishtiaq, Shabina Rahim, Jamshid Abdul-Ghafar, Zubair Ahmad

**Affiliations:** 1 Department of Pathology and Laboratory Medicine, Section of Histopathology, Aga Khan University Hospital, Karachi, Pakistan; 2 Department of Pathology and Clinical Laboratory, French Medical Institute for Mothers and Children (FMIC), Kabul, Afghanistan

**Keywords:** Gliosarcoma, Glioblastoma, Glial, Astrocytic, Mesenchymal, Biphasic, Cerebral hemispheres, Pediatric

## Abstract

**Background:**

Gliosarcoma is a rare variant of IDH- wild type glioblastoma with both glial and mesenchymal differentiation. It accounts for approximately 2% of glioblastomas and has a poor prognosis similar to that of classic glioblastoma. It is seen mostly between 40 and 60 years of age with a mean age over 50 years. Pediatric gliosarcoma is even rarer than gliosarcoma in adults. We describe the clinicopathological features of gliosarcoma in patients under 20 years of age and determine whether there are significant differences from gliosarcoma in adults. We also present detailed review of published literature on pediatric gliosarcoma.

**Methods:**

Slides of gliosarcomas in patients under 20 years of age were reviewed. Clinicopathological features were noted in detail and follow up was obtained.

**Results:**

Eleven cases of gliosarcoma were reported in patients under 20 years of age. Ages ranged from three to 19 years (mean age 13 years). Frontal, parietal and temporal lobes were the commonest locations. Mean and median tumor size was six and five cm respectively. All 11 cases demonstrated the classic biphasic pattern. In 10 cases, glial component was astrocytic and was highlighted on GFAP. Sarcomatous component in most cases resembled fibrosarcoma and was high grade in 72.7%. Glial areas were reticulin poor while sarcomatous areas were reticulin rich. In over 45% cases, bizarre tumor giant cells were seen in the sarcomatous areas. In 1 case, sarcomatous areas showed extensive bone and cartilage formation. Other histologic features included hyalinized blood vessels, hemorrhage, infarction, gemistocytic cells, rhabdoid cells etc. Follow up was available in nine patients, five received chemoradiation post resection while three received radiotherapy only. Prognosis was dismal and eight patients died within one to 14 months following resection.

**Conclusions:**

Gliosarcomas in patients under 20 comprised 13% of all gliosarcomas reported during the study period. Frequency and mean age were higher compared to other published reports. Pathological features were similar to those described in literature. Clinicopathological features and prognosis of pediatric gliosarcomas were similar to adult gliosarcomas.

## Introduction

Gliosarcoma, World Health Organization (WHO) grade IV is a rare variant of Isocitrate Dehydrogenase (IDH)- wild type glioblastoma with both glial and mesenchymal differentiation and accounts for approximately 2% of glioblastomas. It has a poor prognosis similar to that of classic glioblastoma. It is seen mostly between 40 and 60 years of age with a mean age above 50 years, is more common in males and occurs mainly in the cerebral hemispheres with the temporal and frontal lobes being the commonest locations. It is characterized histologically by a biphasic pattern composed of alternating glial and mesenchymal (sarcomatous) areas. Both glial and mesenchymal components represent monoclonal proliferations. The clinical profile, imaging, spread and macroscopic appearance of this variant are similar to classic glioblastoma. It is often superficial and deceptively circumscribed. Pediatric gliosarcoma is even rarer than gliosarcoma in adults [1, 2].

Histologically, the glial component is usually astrocytic (like the classic astrocytic glioblastoma) with anaplastic features. Sarcomatous component usually manifests as a spindle cell sarcoma with nuclear atypia, mitoses and necrosis. The glial areas are reticulin poor while the sarcomatous areas are reticulin rich (highlighted on reticulin stain). On immunohistochemical (IHC) staining, glial areas express glial fibrillary acidic protein (GFAP) while the sarcomatous areas are negative [1].

Pediatric gliosarcoma, as stated above, is even rarer. To the best of our knowledge, 45 cases have been reported in literature [3–36].

Herein, we present a series of 11 cases of gliosarcoma reported in patients under 20 years of age. The aim of this study is to describe the clinicopathological features of gliosarcoma reported in patients under 20 and to determine whether there are significant differences from gliosarcomas occurring in adults. We also present a detailed review of published literature on these extremely rare tumors.

## Methods

The Surgical Pathology files of the Section of Histopathology, Department of Pathology and Laboratory Medicine, Aga Khan University Hospital, were searched for gliosarcomas reported between July 1, 2011 and June 30, 2019. Cases reported in patients under 20 years were identified. Slides of these cases were retrieved and were reviewed by the senior authors. The diagnosis was confirmed. Clinical and pathological features were described in detail. Follow up was obtained through verbal telephonic communication with the parents. Ethical exemption was obtained from the Aga Khan University Ethical Review Committee (ERC). All procedures performed on patient tumor samples in this study were in accordance with the ethical standards of the Institutional ERC and with the 1964 Helsinki declaration and its later amendments or comparable ethical standards. Detailed review of published literature was conducted by the authors.

We systematically searched PubMed, Google Scholar and Web of Science detailed for articles on pediatric gliosarcoma with restriction of ‘original article’ and ‘case reports’. We only included articles with English abstracts. We searched using the Medical Subject Heading (MESH) terms and key words ‘pediatric gliosarcoma’. A total of 34 articles were included. Review articles were not included. We collected demographics, clinicopathological and follow up information. All selected articles were assessed for eligibility. All were found to be eligible. No duplicates were found. All 34 were included in qualitative synthesis.

## Results

**Fig. 1:**
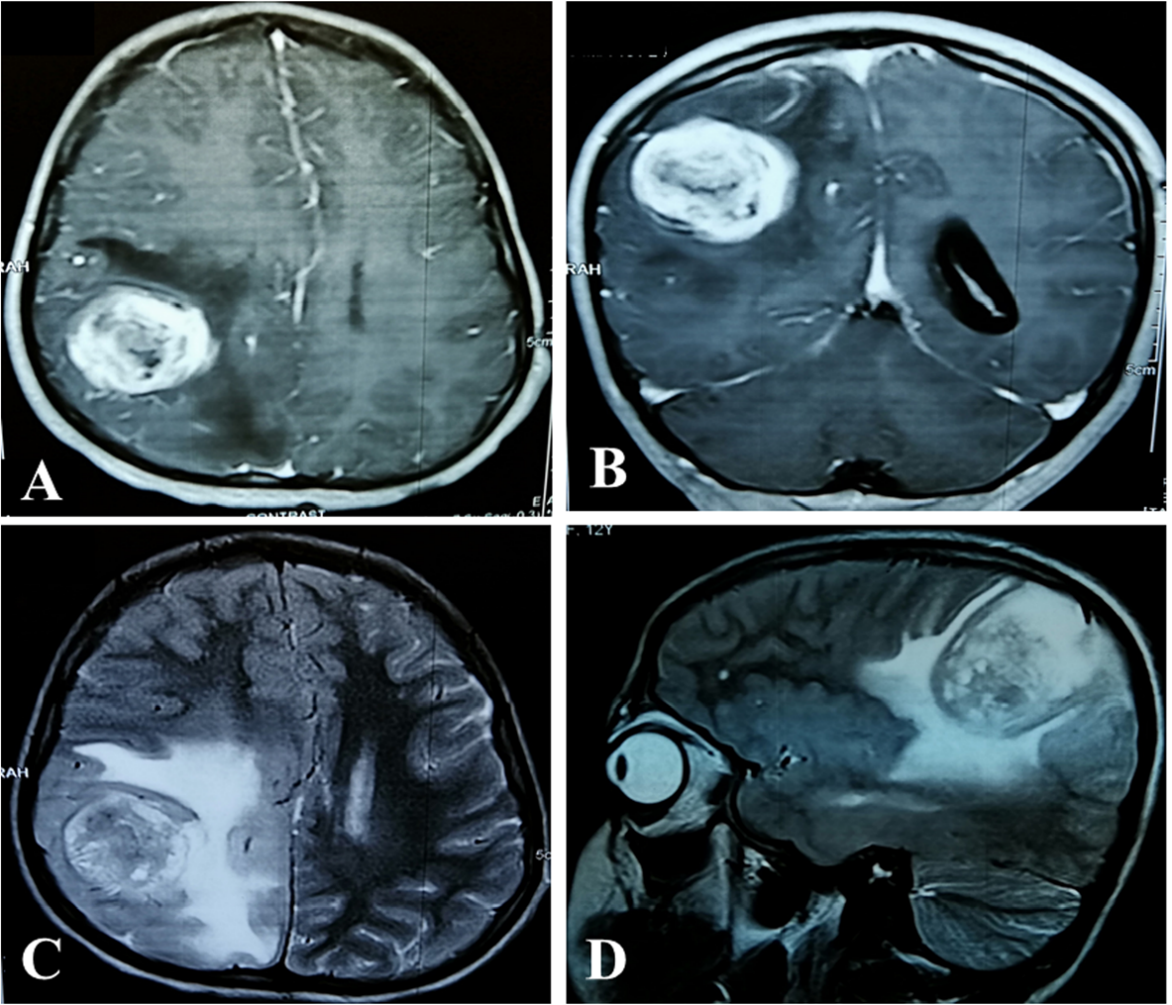
**a** T2WI Axial Image: A well-defined rounded T2WI heterogenous signal intensity mass seen in right parietal lobe with significant perilesional edema. **b** T2WI Sagittal Image: A well-defined rounded T2WI heterogenous signal intensity mass seen in right parietal lobe with significant perilesional edema **c** T1 Axial post-contrast: Avid post contrast enhancing mass with few central hypointensities identified in right posterior parietal lobe and surrounding perilesional edema. **d** T1 coronal post contrast image: Avid post contrast enhancing mass with few central hypointensities identified in right posterior parietal lobe and shows surrounding perilesional edema

**Fig. 2:**
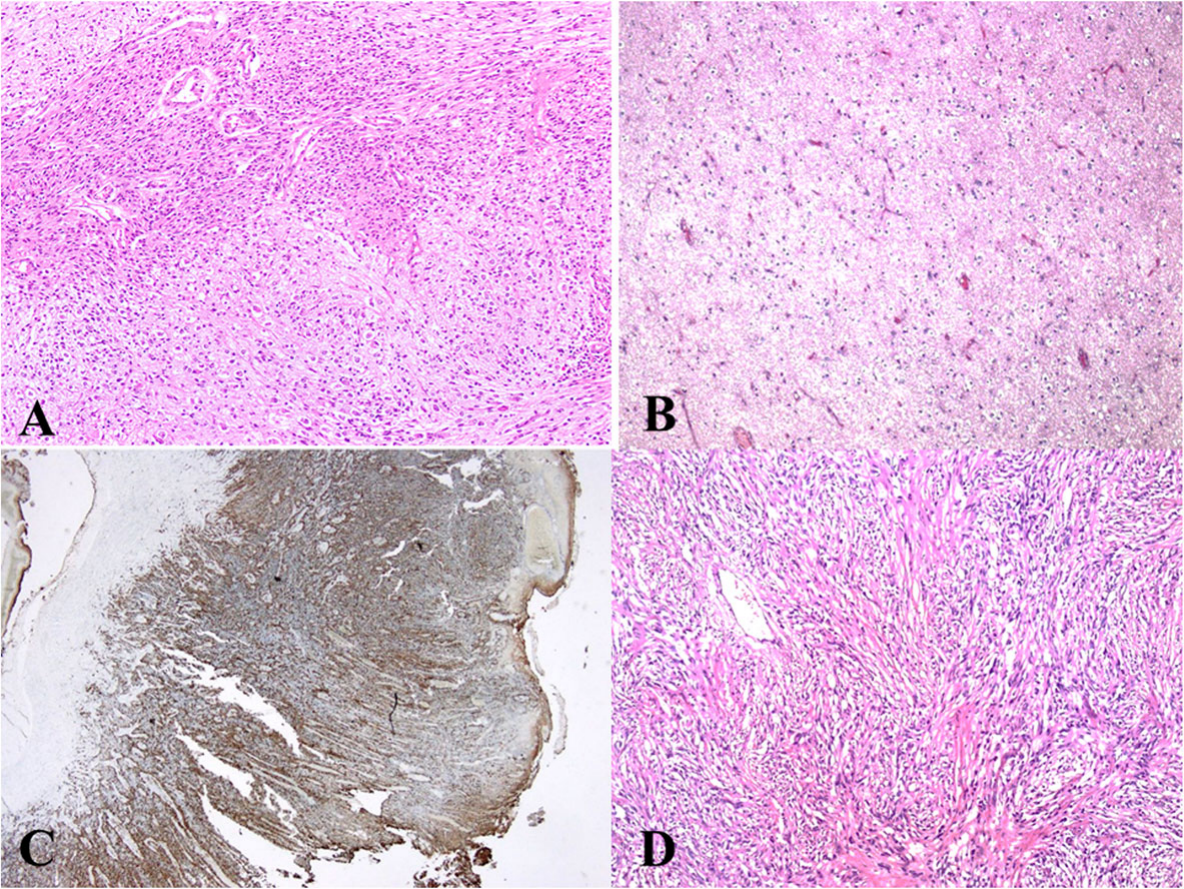
**a** Classic biphasic appearance of gliosarcoma. The mesenchymal elements at top are sharply demarcated from glial component at bottom. **b** Oligodendroglioma as glial component was seen in one case. **c.** GFAP expression in the glial component. **d.** Mesenchymal component appeared as fibrosarcoma in most cases

**Fig. 3:**
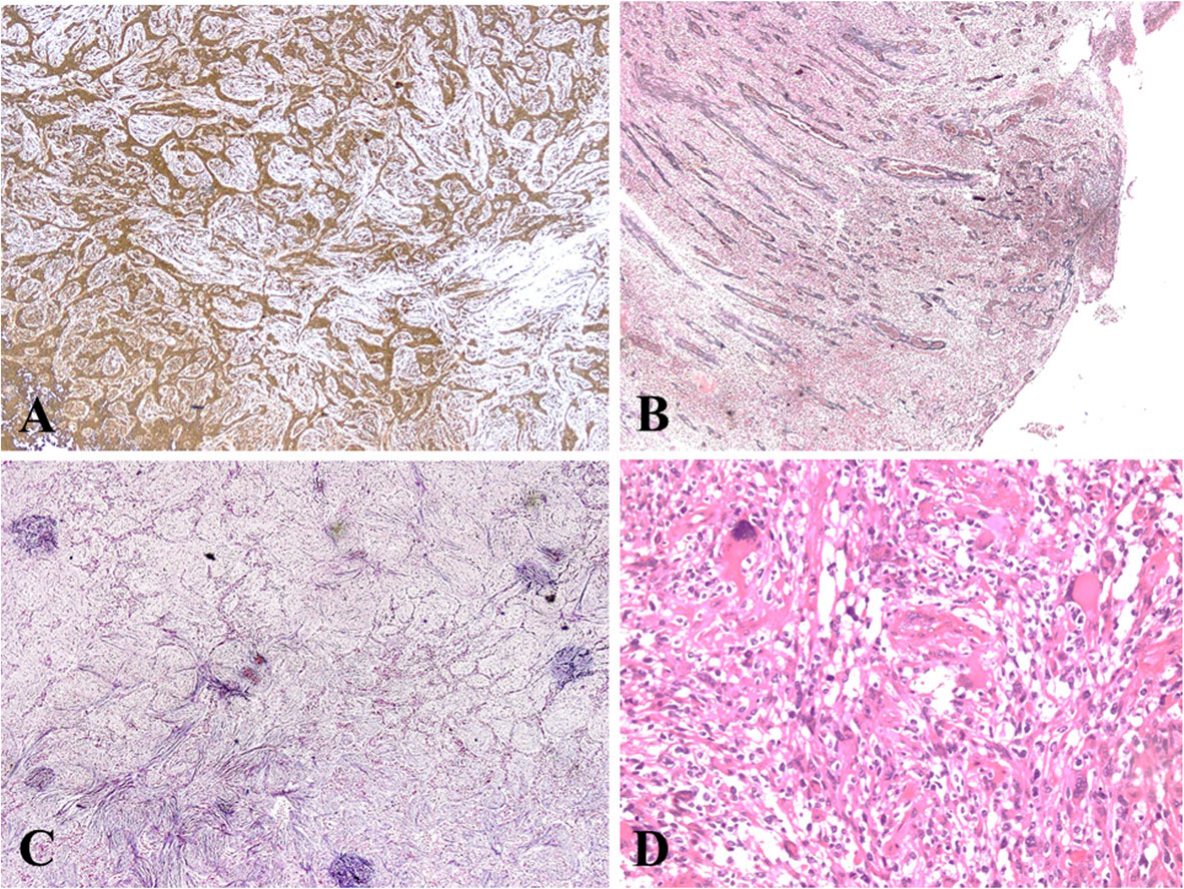
**a.** Vimentin positivity in mesenchymal component. **b** While glial areas are reticulin poor, **c**, mesenchymal component was reticulin rich. **d**. Focal bizarre tumor cells in mesenchymal component

**Fig. 4:**
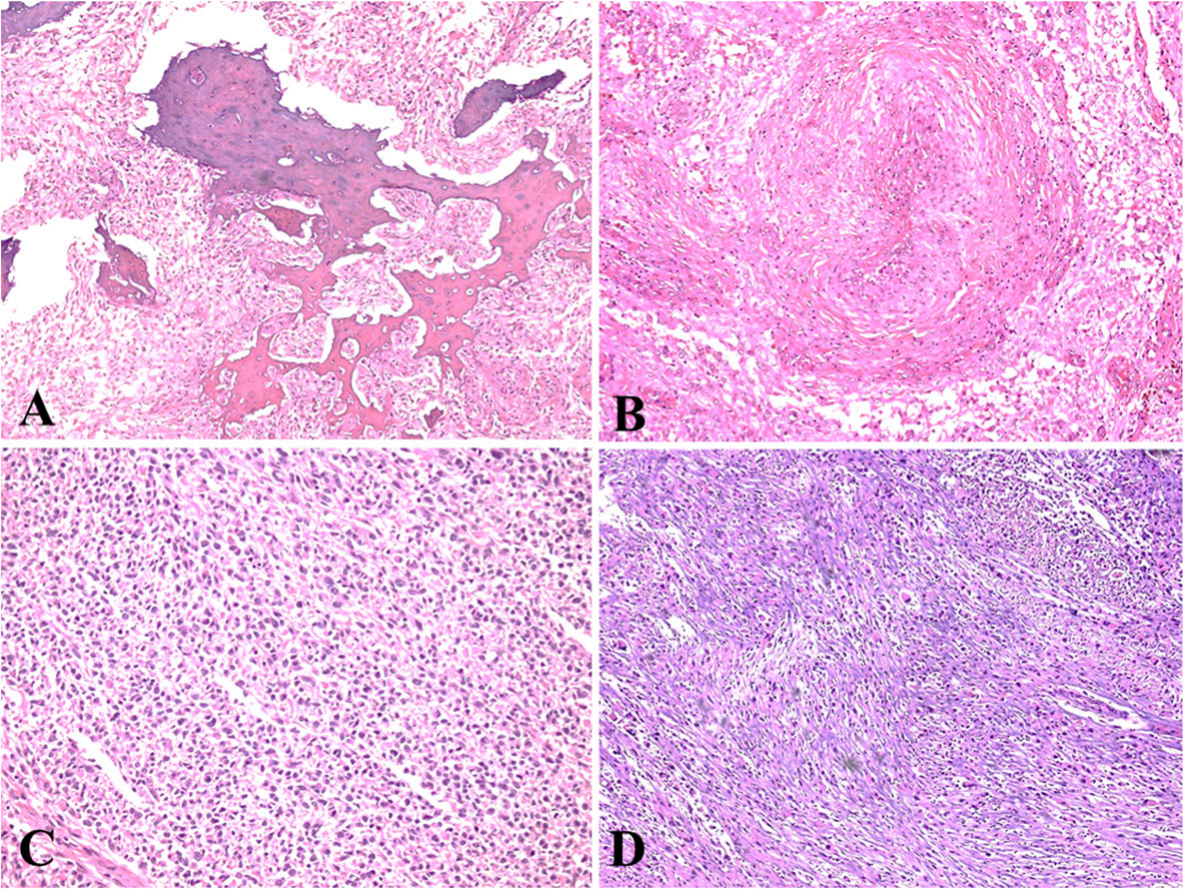
**a.** Trabeculae of lamellar bone in mesenchymal component. **b.** Prominent large vessels were noted in some cases. **c** & **d** Sheets of gemistocytes and rhabdoid cells are focally seen in few cases

During the study period (2011–2019), 84 cases of gliosarcoma were reported. Of these, 11 (31.1%) were reported in patients younger than 20 years of age. Clinicopathological features are summarized in Table 1. Of these 11 patients, 6(54.5%) were males and 5(45.5%) were females. Ages of patients ranged from 3 to 19 years with mean and median age of 13 and 16 years respectively. Four patients (36.4%) were under 10 years of age. Of the 11 cases, 3(27.3%) were located in the frontal and 2(18.2%) in the parietal lobes; one case each was temporal, frontotemporal, temporo-parietal, parieto-occipital, occipital and sellar in location **(**Fig. 1a-d). All cases were received as multiple pieces of tumor tissue ranging from 1.5 cm to 12 cm in aggregate with mean and median size of 6 cm and 5 cm respectively. Gross total resection was apparently not achieved in any of the cases. On histologic examination, all 11 cases demonstrated the classic biphasic pattern **(**Fig. 2a**).** Glial component was astrocytic in 10 cases (90.9%) and oligodendroglial in 1 case (Fig. 2b). Glial component was highlighted in all 11 cases on IHC stain for GFAP (Fig. 2c). Sarcomatous component in most cases manifested as a spindle cell sarcoma resembling fibrosarcoma (Fig. 2d). Sarcomatous component was high grade in 8 (72.7%) cases and low grade in 3 (27.3%) cases. Sarcomatous areas were highlighted on IHC stain for vimentin (Fig. 3a). Glial areas were reticulin poor (Fig. 3b) while sarcomatous areas were reticulin rich (Fig. 3c). In 5 (45.5%) cases, sarcomatous areas showed considerable atypia in the form of bizarre tumor giant cells (Fig. 3d). In 1 case, the sarcomatous component showed additional lines of mesenchymal differentiation in the form of extensive bone and cartilage formation (Fig. 4a). Prominent hyalinized blood vessels (Fig. 4b) were seen in 3 (27.3%) cases while hemorrhage and infarction were noted in 2 cases. Gemistocytic (Fig. 4c) and rhabdoid (Fig. 4d) cells were seen in 1 and 2 cases respectively. Follow up was available in 9 cases. Of these 9 patients, 5 received both chemotherapy and radiotherapy post-surgery while 3 received radiotherapy only. One patient did not receive either chemotherapy or radiotherapy. Of these 9 patients, 8 died within 1 month to 14 months following surgery while the 9th patient was alive 6 months post-surgery.

**Table 1:** Clinicopathological features of pediatric gliosarcomas (*n* = 11)

S.Num	Age/Sex	Site	Tumor size in biopsy (cm)	Chemotherapy	Radiotherapy	Survival following surgery
1	12/F	Right cerebral cortex	2 x 2 x 1.5	Received	Received	Alive 6 months following resection
2	14/F	Right parietal lobe	NK	Received	Received	DOD 4 months following resection
3	15/F	Sellar & suprasellar	6.4 × 6.1 × 4	Not received	Received	DOD 1 month following resection
4	18/M	Left parieto-occipital lobe	3 x 2 x 1.5	Received	Received	DOD 14 months after resection
5	3/M	Fronto-parietal lobe	10 x 7 x 5	Not received	Received	DOD 6 months after resection
6	16/F	Left frontal lobe	5 × 1.9 × 1.5	Received	Received	DOD 3 months after resection
7	8/F	Left temporal lobe	5 x 4 x 3	Not received	Received	DOD 1 month following resection
8	19/M	Left parietal lobe	7.3 × 5.5 × 2	Not received	Not received	DOD 8 months following resection
9	5/M	Right frontal lobe	12 x 11 x 3	Received	Received	DOD 3 months following resection
10	10/M	Frontal lobe	1.5 × 1.5 × 0.3	NK	NK	Lost to follow up
11	15/M	Left parietal lobe	5 × 3.5 × 2 cm	NK	NK	Lost to follow up

*DOD* died of disease; *NK* not known

## Discussion

Pediatric gliosarcomas are even rarer than adult gliosarcomas. Clinical and morphological features are similar to adult gliosarcomas. However, they may mimic more common tumors on radiological and histological examination. The clinicopathological features and differential diagnostic consideration are discussed. Published literature is reviewed.

On radiological examination, the relative discreteness of these tumors may mimic meningioma [37, 38]. Similarly, on gross examination, the appearance of a firm well-circumscribed mass with attachment to dura may be mistaken with a meningioma or metastases, although such confusion is more likely in adults rather than in the pediatric age group [1]. On histological examination, the diagnosis in typical cases even in pediatric age group is straightforward when the classic biphasic pattern is well developed. However, the sarcomatous areas in both adult and pediatric gliosarcoma can resemble fibrosarcoma. Some cases may show other types of mesenchymal differentiation such as bone and cartilage formation (resembling osteo-or chondrosarcoma), smooth and striated muscle differentiation (resembling leiomyosarcoma and rhabdomyosarcoma), lipomatous differentiation (resembling liposarcoma) and primitive neural differentiation. Variable mesenchymal differentiation was seen in our series and additional mesenchymal differentiation in the form of bone and cartilage formation was seen in one case. Similarly, adenoid (epithelial) differentiation may be seen in gliosarcoma in all age groups and such areas may resemble carcinoma resulting in misdiagnosis. Squamous metaplasia may be seen and can be mistaken for squamous cell carcinoma. The presence of gemistocytic and rhabdoid cells may lead to erroneous diagnosis of gemistocytic astrocytoma and atypical teratoid/rhabdoid tumor in the pediatric age group. Gemistocytic and rhabdoid cells were seen in one and two cases respectively in our series. In pediatric patients, germ cell tumors (such as germinoma and teratoma) should also be considered in the differential diagnosis. Gliosarcoma in pediatric patients can especially be confused with teratoma if bone, cartilage or other mesenchymal components are present [22, 39–43]. Desmoplastic infantile astrocytoma (DIA) is another rare tumor which can be confused with gliosarcoma in the pediatric age group. However, DIA is a slow growing, WHO grade I tumor which typically occurs in infants as a large cystic mass in the supratentorial cerebral cortex and meninges and is often attached to the dura. Microscopically, it is composed of a prominent desmoplastic stroma in which streams of neoplastic astrocytes are seen. Mitotic activity and necrosis are uncommon and ki67 index is usually < 2% [1].

At the molecular level, gliosarcomas including those in the pediatric age group demonstrate Phosphatase and Tensin homolog (PTEN) and TP53 mutations and Cyclin-dependent kinase inhibitor 2A (CDKN2A) deletions. Epidermal growth factor receptor (EGFR) amplification is infrequent. Except for the last, their genetic profile is similar to that of IDH-wild type glioblastoma. Gains on chromosome 7 are seen in 75% cases while losses on chromosome 10 are seen in 35% [44]. In a study of adult gliosarcomas by Smith et al., these tumors were primarily 0–6-Methylguanine-DNA-Methyltransferase (MGMT) unmethylated (87.5%), IDH-1 preserved (100%) and EGFR wild type (100%). A 2019 study by Lowder et al. demonstrated that the most frequent alteration was copy number loss comprising 57% of total copy number changes and far exceeding the number of copy number gains (26.2%), amplifications and loss of heterozygosity events. Chromosomes 9 and 10 showed the highest number of losses while the majority of copy number gains were seen on chromosome 7 [45, 46]. Recently, Graham et al. reported a gliosarcoma in an eleven-year-old girl and a twelve-year-old boy. The latter had neurofibromatosis type 1 (NF1), the first reported case of pediatric gliosarcoma in a child with NF1. Whole-exome sequencing showed higher mutational burden in the patient without NF. NF1 patient survived without progression while patient without NF1 died of disease [36].

Occasional case reports documenting pediatric gliosarcomas in locations other than the cerebral hemispheres have been published. Neelima et al. reported a case occurring in the thalamus [21]. A case of pediatric gliosarcoma associated with NF1 was recently reported by Dogan et al. [33]. Granados et al. reported a pineal gliosarcoma in a five-year-old girl, the first reported case in this unusual location [29].

Various studies have emphasized the importance of gross total resection in achieving relatively better prognosis [23, 32]. Studies have shown that subtotal resection is the most important variable in the dismal prognosis associated with pediatric gliosarcomas in most cases [31]. However, a number of studies have shown that prognosis is dismal even in cases where apparent gross total resection was achieved and in spite of aggressive chemo and radiotherapy post resection [15, 20, 26], median overall survival and event free survival have been only a few months, mostly under a year [15, 20, 37]. Few studies, however, have reported better prognosis and long-term survival with aggressive treatment (gross total resection, chemotherapy and radiotherapy [18, 24, 47]. However, overall, pediatric gliosarcomas share a dismal prognosis with adult gliosarcoma. Thus, although a longer survival has been reported in a few cases, the majority of patients demonstrate an extremely poor prognosis with early recurrence and death within a few months after surgery even after apparent gross total resection and aggressive post-surgical chemo and radiotherapy [26, 31]. This was true for our cases except for two patients who survived for 14 months and 2 years respectively post resection. Both these patients received chemo and radiotherapy. Findings of comprehensive literature review are summarized in Table 2.

**Table 2:** Summary of all cases of pediatric gliosarcoma published previously by literature search (*n* = 42)

S.No	Ref	Year of publication	Number of cases	Age (years)	Gender (Male/Female)	Location	Extent of resection (Total/partial)	Post-resection radio/chemotherapy	Follow up
1	Goldstein et al. [3]	1981	1	0	Female	Left cerebral hemisphere (diffuse widespread involvement)	–	–	–
2	McKeewer et al. [4]	1984	1	18	Female	Occipital lobe	Subtotal	Radiotherapy	Died 12 months after resection
3	Cerame et al. [5]	1985	1	11	Female	Left posterior temporo-parietal lobes	Total	Radiotherapy	Died 1 month after resection (developed thoracic metastases at 2 weeks)
4	Lee et al. [6]	1985	2	12	Male	Right frontal lobe	Total	Radiotherapy (had Hodgkin lymphoma seven years ago)	Alive at 16 months after resection
				14	Female	Both frontal lobes & corpus callosum	Partial	Partial	Died 3 months after resection
5	Takaue et al. [7]	1986	1	11	Male	Right fronto-parietal lobes	Total	Radiotherapy	Alive at 25 months after resection (had Hodgkin lymphoma seen years ago)
6	Chadduck et al. [8]	1987	1	2	Male	Right cerebral cortex (diffuse widespread involvement)	Partial	Not given	–
7	Radkowski et al. [9]	1988	1	0	Male	Right temporal lobe	–	–	Alive at 21 months after resection
8	Ono et al. [10]	1990	1	0	Female	Left temporo-parietal lobes & basal ganglia	Total	Radiotherapy	Alive at 34 months after resection
9	Kaschten et al. [11]	1995	1	13	Male	Right temporo-parieto-occipital lobes	Total	Radiotherapy	Died 13 months after resection
10	Lach et al. [12]	1996	1	18	Male	Right frontal lobe	Partial	Not given	Died 5 months after resection (had diffuse astrocytoma, WHO grade II 10 years ago)
11	Kepes et al. [13]	1996	1	19	Female	Left parieto-occipital lobes	–	Not given	Died 7 months after resection (had recurrent ependymoma, WHO grade II 29 months ago; irradiated)
12	Rizk et al. [14]	2000	1	0		Left temporo-parieto-occipital lobes	Total	Not given	Perioperative death
13	Okami et al. [15]	2002	1	2	Male	Left frontal lobe	Total	Radiotherapy	Died 3 months after resection (relapsed in one month)
14	Malde et al. [16]	2004	1	21	Female	Left frontal lobe	Total	Radiotherapy	Alive at 6 months after resection (Medulloblastoma eight years before, radiation)
15	Deb et al. [17]	2006	1	18	Male	Right frontal lobe	Decompression	Irradiation given for giant cell glioblastoma (transformation to gliosarcoma)	Alive at 1 month after decompression
16	Salvati et al. [18]	2006	3	15	Female	Right temporal lobe	Total	Not given	Died 5 months after resection
				13	Female	Midline tumor-parieto-occipital lobes reaching corpus callosum	Subtotal	Radiotherapy	Alive at 9 months after resection
				16	Male	Parasagittal frontal	Total	Radiotherapy	Alive at 24 months after resection (Hemangioblastoma 10 years ago, radiation given)
17	Hocwald et al. [19]	2009	1	0 (congenital)	Male	Left anterior cerebral hemisphere	None	Not given	Bulging anterior fontanelle. Non-responsive at birth. Intensive care was withdrawn after consultation with parents. Baby died at 1 day of age.
18	Karremann et al. [20]	2010	4	8	Female	Right temporal lobe	Total	Radiotherapy	Died 6 months after resection
				10	Male	Right temporal lobe & lateral ventricle	Total	Radiotherapy	Died 18 months after resection
				6	Male	Right fronto-parietal lobes, corpus callosum	Partial	Radiotherapy	Died 4 months after resection
				9	Male	Right mesencephalon	Partial	Radiotherapy	Alive at 14 months after resection
19	Neelima et al. [21]	2012	1	11	Male	Thalamus	Near total	–	–
20	Ravisanker et al. [22]	2012	1	11	Male	Right temporo-parietal cortex	Total	Radiotherapy, Temozolomide	Alive
21	Moscote-Salazara et al. [23]	2014	1	4	Male	–	–	Radiotherapy	Alive
22	Martin et al. [24]	2014	1	11	Male	–	Near total	Radio & chemotherapy	Alive at 34 months after resection
23	Burzyuski et al. [25]	2014	1	9	Male	Pons	Subtotal	Chemotherapy	Alive at > 13 years*
24	Savant et al. [26]	2015	1	5	Female	Left parieto-occipital lobe	Total	Chemotherapy	Died 9 months after resection
25	Mallick et al. [27]	2015	5	7	Female	–	Total	Radiotherapy, Temozolomide	Progressed 13 months after resection
				–	Male	–	Subtotal	–	Lost to follow up
				–	Female	–	Total	Radiotherapy, Temozolomide	Alive at 3.5 years after resection
				–	Female	–	Total	Radiotherapy, Temozolomide	Alive at 2 years after resection
				19	Female		Total	Radiotherapy, Temozolomide	Progressed 43 months after resection
26	Meena et al. [28]	2016	1	12	Female	Right parieto-occipital lobe	Total	Radiotherapy	–
27	Granados et al. [29]	2017	1	5	Female	Pineal	–	Radio & chemotherapy	Metastatic dissemination
28	Yao et al. [30]	2017	1	6	Female	Cervical spine (C1-C6)	–	Radiotherapy	Died 6 months after resection
29	Dutta et al. [31]	2018	1	8	–	Parieto-occipital lobe	Subtotal	Radio & chemotherapy	Tumor recurred. Repeat surgery. Died
30	Bouali et al. [32]	2020	1	0 (5 mos)	Male	Right frontal lobe	Total	Not given	Alive at 17 months after resection with midline left frontal residual tumor
31	Dogan et al. [33]	2020	1	3	Male	Left parietal lobe extending to the vertex	Palliative resection	Not received	Alive
32	Jeng & Reynolds [34]	2020	1	12	Male	Right frontal lobe	Partial	Radio & chemotherapy	Recurred 8 months after resection. Alive **
33	Bukhari et al. [35]	2020	1	12	Male	Occipital lobe	Subtotal	–	Recurrence 2 years after resection. Re-resection done, then lost to follow up
34	Graham et al. [36]	2020	2	12	Male	Right frontal lobe	Total	Radio & chemotherapy	No progression after 68 months
				12	Female	Right temporal lobe	Near total	Radio & chemotherapy	Recurrence after 2 years; Died 54 months after diagnosis

Previous history of low- grade astrocytoma treated with subtotal resection, standard radiotherapy, chemotherapy and gamma knife procedure

** Initially glioblastoma which was resected and treated with chemo & radiotherapy

Mallick et al. published a series of five cases of pediatric gliosarcoma and investigated the value of concurrent and adjuvant temozolamide in the treatment of these tumors. They showed that temozolamide is well tolerated by pediatric patients and survival data with temozolamide therapy was encouraging. The two-year progression free and overall survival rates were 44.2 and 62.9%, respectively [27].

## Limitations

1. Follow up was available in only 9 out of 11 cases
2. Molecular workup was not performed.

## Conclusions

Pediatric gliosarcomas are extremely rare. Clinicopathological features of pediatric gliosarcoma are similar to adult gliosarcoma. However, pediatric gliosarcomas may mimic more common tumors on radiological and histological examination. On histological examination, gliosarcomas may sometimes mimic sarcoma and carcinoma if specific mesenchymal and glandular differentiation is present. In pediatric age group, osteosarcoma, fibrosarcoma, teratoma, and atypical teratoid/rhabdoid tumor should be excluded. Like their adult counterparts, pediatric gliosarcomas have a dismal prognosis in spite of aggressive chemoradiation. Slightly better survival times have been demonstrated in some studies with gross total resection although other studies have shown extremely poor survival even with apparent gross total resection.

## Data Availability

Data and materials of this work are available from the corresponding author on reasonable request.

## Abbreviations

WHO: World Health Organization
IHC: Immunohistochemical
GFAP: Glial fibrillary acidic protein
ERC: Ethical Review Committee
OS: Overall survival
PFS: Progression free survival
DIA: Desmoplastic infantile astrocytoma
PTEN: Phosphatase and Tensin homolog
CDKN2A: Cyclin-dependent kinase inhibitor 2A
EGFR: Epidermal growth factor receptor
IDH: Isocitrate Dehydrogenase
MGMT: Methylguanine-DNA-Methyltransferase
NF1: Neurofibromatosis type 1
MESH: Medical Subject Heading
